# Automated analysis of bone-conduction cortical auditory evoked potential in normal-hearing neonates

**DOI:** 10.1016/j.bjorl.2019.09.007

**Published:** 2019-10-31

**Authors:** Daniela Soares de Brito, Alessandra Spada Durante

**Affiliations:** a Faculdade de Ciências Médicas da Santa Casa de São Paulo, São Paulo, SP, Brazil; b Universidade de São Paulo, São Paulo, SP, Brazil

**Keywords:** Audiology, Electrophysiology, Auditory evoked potentials, Neonates, Bone conduction

## Abstract

**Introduction:**

The cortical auditory evoked potential allows the possibility of objectively evaluating the entire auditory system, which is desirable in the pediatric population. Bone conduction auditory stimulation is recommended in the differential diagnosis of conductive hearing loss. However, there are not many studies of cortical auditory evoked potential using bone conduction.

**Objective:**

The aim of this study was to characterize the response of cortical auditory evoked potential through bone conduction in normal-hearing neonates using an automated response analysis equipment.

**Methods:**

This study included 30 normal-hearing neonates, without risk factors for hearing loss. The equipment used was the HEARlab automated response analysis and the cortical responses were evaluated at the frequencies of 500–4000 Hz through bone conduction, at intensity ranging from 0 to 60 dBnHL. The latencies and amplitudes were manually marked by experienced judges.

**Results:**

Cortical auditory evoked potential responses were detected in 100% of the evaluated subjects and there was no difference regarding the cortical response of the neonates in relation to the variables of gender, ear and masking use. At an intensity of 60 dBnHL for the frequencies of 500, 1000, 2000 and 4000 Hz the latencies were 234; 241; 239 and 253 ms and the amplitudes were 15.6; 8.4; 6.2; 6.3 μV. The mean thresholds were 23.6; 28; 31 and 33.1 dBnHL, respectively.

**Conclusion:**

It was possible to measure the cortical auditory evoked potential response in the neonatal population using bone vibrator as sound transducer and to draw the profile of the cortical auditory evoked potential latencies and amplitudes by frequencies at the intensity of 60 dBnHL and at the threshold.

## Introduction

The clinical need for efficient and reliable methods to assess hearing in the pediatric population has resulted in a large number of investigations and advances in the studies of cortical auditory evoked potentials (CAEP), which has several advantages since it evaluates the entire auditory system. The CAEP allows access to the function of central auditory structures and verifies auditory maturation.^1^, ^2^

The Australian governmental institute, the National Acoustic Laboratory (NAL), has developed a CAEP response capture device, called the HEARlab System, which can capture the CAEP while reducing noise and artifacts, detect and perform an automated statistical analysis of responses, which obviates the need for interpretation of responses based on an examiner's experience and subjectivity.^3^ It can also be used with different transducers: air-in earphones, bone conduction headphones or speakers for free field testing, which makes its applicability comprehensive and favorable to several diagnostic needs.

The contribution of automated capture of responses when exploring these potentials in the pediatric population indicates the effectiveness of Hotelling's T2, which detects the response of the CAEP at a rate equal to that of an experienced examiner in children with normal hearing,^4^ neonates,^5^, ^6^ users of individual sound amplifier device (ISAD)^7^ and children with cochlear implants.^8^ A recent study with automated detection used BC to verify the impact of chronic unilateral conductive hearing loss on CAEP and found significantly higher P1 ‒ N1 and N1 ‒ P2 amplitudes in hearing impaired individuals.^9^ These results are the first to provide direct evidence of increased amplitude of the neural response in the adult human auditory cortex in the presence of unilateral conductive hearing loss.

The importance of bone conduction evaluation is maximal in small children due to the high occurrence of otitis and also in the presence of external and/or middle ear malformations. Although there is a recommendation for bone conduction electrophysiological investigation in the audiological diagnosis of the pediatric population,^10^, ^11^ few studies in the literature have reported the use of bone conduction (BC) to estimate short latency electrophysiological responses^12^, ^13^, ^14^ and there are controversies about the protocol, especially regarding the positioning and force of the bone transducer and the use of contralateral masking.^15^, ^16^

Thus, the present study aimed to characterize the response of CAEP by BC in neonates with normal hearing, analyzing the P1 component latencies and amplitudes at 60 dBnHL intensities and cortical threshold at 500, 1000, 2000 and 4000 Hz frequencies, using automated response analysis equipment.

## Methods

This is an observational, prospective, descriptive, cross-sectional study focused on diagnosis. It was approved by the Human Research Ethics Committee under N. 951.829. The parents/guardians of the study participants were informed about the purpose of the study and, upon agreeing to participate, signed the free and informed consent form.

Neonates born at the institution's maternity hospital, from June 2016 to May 2018, were evaluated according to the following inclusion criteria: neonates aged 6 to 28 days old, with positive result at the transient evoked otoacoustic emissions (TEOAE) during neonatal hearing screening and no hearing impairment risk indicator (HIRI) according to the criteria of the Joint Committee on Infant Hearing^17^ (Annex III); head circumference > 32 cm (WHO, 2016); acoustic immittance with tympanometric curve type A and passing result in the automated auditory brainstem response (AABR) (expanded neonatal hearing screening).

To perform the expanded neonatal hearing screening through immittance tests, TEOAE and AABR, the Interacoustics® Titan equipment was used. For tympanometry, pump speed with an initial pressure of +200 daPa and stop pressure of −400 daPa was used, with pre-defined age category as newborns, using the frequency of 1000 Hz. For TEOAE measurement, a nonlinear broadband (frequency range 500–5000 Hz) click stimulus was used, with click intensity at 80 dB SPL small. For AABR recording, the CE Chirp® stimuli were presented at a repetition rate of 90 Hz, with alternating polarity at the intensity of 35 dBnHL. This recording has an automated response detection method, which uses the “q-sample test” and Bayesian Weighting.

The equipment used to record the CAEP was the HEARlab in the “Cortical Threshold Evaluation” (CTE) module, which allows the detection of cortical responses to specific frequency sound stimuli, automatically presented at frequencies from 500 to 4000 Hz. Acoustic stimuli range from 0 to 110 dBnHL and were presented by bone conduction. Alternating polarity was used, with an interstimulus interval of 1.125 ms and speed of 0.5 Hz, with a total duration of 40 ms. There was a cosine rise of 10 ms and plateau-fall time of 20 ms (Table 1).

**Table 1:** Protocol for CAEP registration on HearLab equipment.

Polarity	Alternating
Interstimulus range	1.125 ms
Velocity	0.5 Hz
Type of stimulus	Tone-burst at frequencies: 0.5; 1; 2; 4 kHz
Total duration	40 ms
Cosine increase	10 ms
Time of fall	20 ms
Detection of P1	Automated (Hotelling's T2 statistic test) Present P1 *p* < 0.05
Time of analysis	600 ms
Recording channels	2 channels
Analogic amplification	1.210×
High pass filter	12 dB/octaves of 4000 Hz
Low pass filter	06 dB/octaves below 3000 Hz
Artifact rejection	Voltage difference of active-reference electrodes
Number of responses	50‒200
Stimulator	B71 Bone Vibrator and EAR3A Insertion Earphone
Electrodes	Fpz; CZ; M1 or M2
Masking stimulus	Narrow Band Noise
Masking intensity	S/R -30 dBSPL

The equipment was made available by the Speech-Language Pathology Clinic-School of ISCMSP (Irmandade da Santa Casa de Misericórdia de São Paulo) and complies with the standards: ANSI S3, 6–1989; ANSI S3, 431992; IE 645-1 (1992); IEC 645-2 (1993); UL 544. The equipment was calibrated according to the technical criteria established by the manufacturers.

### Procedure

Initially, still in the maternity ward of ISCMSP, the participant characterization protocol was filled out to obtain overall and hearing health information to verify the inclusion criteria. Once the inclusion criteria were met, the parents/guardians were invited to participate in the research and, if they agreed, a date was scheduled to carry out the research protocol at the Speech-Language Pathology Clinic of ISCMSP, within two to four weeks.

The tests were performed at the Speech-Language Pathology Clinic of ISCMSP in an acoustically treated room. The newborn’s parent/guardian was accommodated in a comfortable armchair and instructed to keep the newborn on their lap.

**Figure 1:**
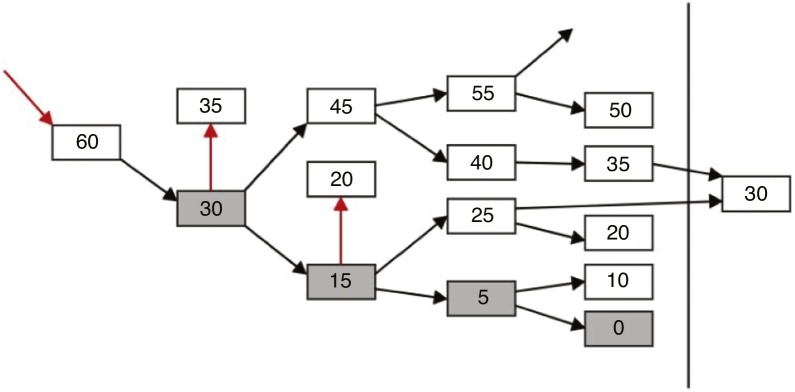
Proposed modification of the threshold research strategy protocol by Van Dun et al.^18^

The skin of the newborns was prepared with gauze to promote electrode fixation. When necessary, Nuprep abrasive paste was used to promote skin cleansing and ensure impedance of less than 5 kOhms for all electrodes. Disposable electrodes were fixed at Fpz (ground), Cz (active) and M1 or M2 (reference) positions using Ten20 electrolytic paste and hypoallergenic adhesive tape. The stimuli were presented through bone conduction (radio ear B71) fixed on the newborn’s mastoid with an elastic band (5 cm wide, self-adherent, Brazil 3 M) with a force of 400 g, at frequencies of 500, 1000, 2000 and 4000 Hz, with intensity ranging from 0 to 60 dBnHL in only one ear, chosen at random. Frequency presentation at the various intensities was based on the adaptation of the stimulus decision protocol proposed by Van Dun et al.^18^ (Fig. 1). The maximum intensity of 60 dBnHL was used to avoid artifacts and to obtain greater reliability for the CAEP responses and, thus, verify the integrity of the auditory pathway. Then we sought the minimum response threshold by first testing the intensity of 30 dBnHL at all frequencies and, when verifying the presence of the response, the stimulus was presented sequentially at 15 dBnHL, 5 dBnHL and 0 dBnHL, always intercalating the frequencies. In the absence of response, the stimuli were increased by 5 dBnHL until the electrophysiological threshold response was recorded. At each lack of response, the stimulus was repeated at the same intensity to confirm its absence.

The analysis for the presence or absence of responses and the respective CAEP thresholds was automatically generated by the equipment. The P1 component latency and amplitude were manually recorded by three observers. The observers are speech-language pathologists experienced in electrophysiology who recorded the CAEP latencies and amplitudes in the results without prior identification of other recordings.

The latencies and amplitudes of the CAEP responses by BC were analyzed for each frequency (500, 1000, 2000, 4000 Hz) at 60 dBnHL intensity and at the electrophysiological threshold.

To increase the reliability of the present research, two complementary studies (masking and CAEP by air conduction) were performed in part of the sample and the results were compared with the total sample.

For the study of the masking effect, during the CAEP measurement, the contralateral masking with narrow band noise was applied. First, the maximum intensity of 60 dBnHL was tested. Then, the 60 dBnHL intensity with contralateral masking in the signal-noise ratio of −30 dB SPL was used again, and the responses were obtained for each frequency. After the tests were recorded by the observers, a comparative study of the responses with and without the use of masking was performed to evaluate latencies and amplitudes.

For the study of CAEP by air conduction (AC), the Oliveira et al. protocol was applied^6^ to search for P1 latencies and amplitudes at 80 dBnHL in AC. Then, the protocol of the present study was carried out, which sought the CAEP latencies and amplitudes by BC at 60 dBnHL. After the tests were recorded by the observers, a comparative study of the amplitude and latency responses between the cortical responses obtained by AC and BC was performed.

Statistical analysis of the data set was performed by the FCMSCSP (Faculdade de Ciências Médicas da Santa Casa de São Paulo). The descriptive exploratory analysis of data was used, applying the measures of Central Tendency and Pearson’s Correlation, while the Wilcoxon, Mann-Whitney and Friedman tests were used for the inferential analysis. The descriptive level was emphasized in all tests, and the significance level of 0.05% or 5% was used to reject the null hypothesis.

The intraclass correlation coefficient statistical test was also used to analyze the agreement between the observers regarding the latency and amplitude variables of the CAEP.

## Results

Ninety-seven neonates, born at the ISCMSP maternity hospital, were invited to constitute the sample, but only 35 accepted, and of these, 5 were excluded because it was not possible to complete the study protocol. The study sample comprised 30 neonates, 10 females and 20 males, with a mean gestational age of 38.89 weeks, with a postpartum mean age of 11.9 days, and a mean head circumference of 33. 57 cm. Fifteen right ears and 15 left ears were tested.

The average test time duration was 80 min, with the shortest lasting 36 min and the longest, 95 min. This time varied due to the subject status and whether masking was used or not. Regarding the test time that included the CAEP-AC test, the average test time was 134 min, with shortest being 120 min and the longest, 144 min.

In the expanded screening, all newborns had present TEOAE, tympanometric curve type ‘A’ and “pass” result in the AABR.

For the study of masking effect, we performed the comparative analysis of P1 at frequencies from 500 to 4000 Hz, at the intensity of 60 dBnHL, with and without masking, and no statistical difference was found in P1 wave latency and amplitude for all frequencies.

For the study of the comparison of latencies and amplitudes between the air conduction (AC) and the bone conduction (BC) CAEP, the P1 component was compared, at the frequencies from 500 to 4000 Hz, at the intensity of 80 dBnHL for AC and 60 dBnHL for BC, and no statistically significant difference was found in P1 wave latency and amplitude for all frequencies.

In the comparative analysis of the P1 component latency and amplitude at frequencies from 500 to 4000 Hz between the ears at the intensity of 60 dBnHL and at the cortical threshold, there was no statistically significant difference (*p* > 0.05), emphasizing that this analysis was performed between ears of different subjects.

The results of the comparative analysis of the P1 latency and amplitude component at the frequencies of 500, 1000, 2000 and 4000 Hz at 60 dBnHL and the threshold, according to gender, showed no significant differences.

Table 2 shows the latency values ​​at the maximum intensity tested, that is, 60 dBnHL, at frequencies of 500, 1000, 2000 and 4000 Hz. The values ​​found show that there was no statistically significant difference among the frequencies.

**Table 2:** Description of P1 latencies by frequency at 60 dBnHL.

	Latency					
Frequency (Hz)	Mean	Median	SD	Minimum	Maximum	*p*^a^
500	234	238	42	109	344	0.207
1k	241	238	60	138	419	
2k	239	230	58	142	423	
4k	253	250	43	195	350	

a Friedman’s test.

Table 3 shows the mean amplitude values ​​at the maximum intensity tested, i.e., 60 dBnHL, from 15.6 μV to 500 Hz; 8.4 μV for 1000 Hz; 6.2 μV for 2000 Hz and 6.3 μV for 4000 Hz. A statistically significant difference was found for the tested frequencies, showing *p* = 0.001 in a multivariate analysis by Friedman’s test. To verify the difference between the frequency pairs, the Wilcoxon test was performed, which showed a significant difference at the frequency of 500 Hz in relation to the other tested frequencies (*p* = 0.001). The frequencies of 1000, 2000 and 4000 Hz showed no significant differences among them.

**Table 3:** Descriptive analysis of P1 amplitudes by frequency at the intensity of 60 dBnHL.

Frequency (Hz)	Amplitude					
	Mean	Median	SD	Minimum	Maximum	*p*^a^
500	15.6	12.5	9.3	3.5	40.7	<0.001
1k	8.4	7.4	7.0	0.2	24.9	
2k	6.2	5.0	4.0	0.9	15.8	
4k	6.3	5.3	4.2	1.1	17.3	

a Friedman’s test.

**Figure 2:**
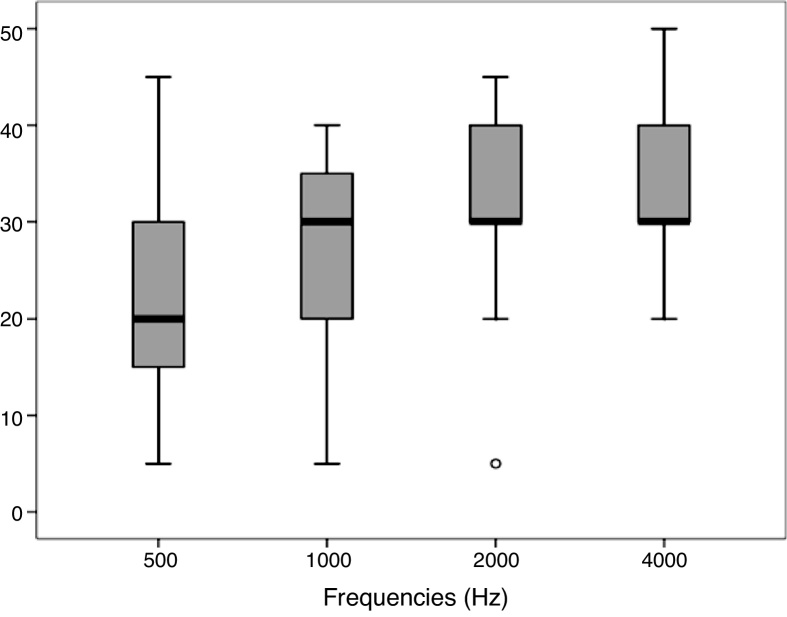
Description of electrophysiological thresholds for the frequencies of 500, 1000, 2000 and 4000 Hz.

In the analysis of the electrophysiological thresholds obtained in the CAEP by BC, it was verified that the thresholds vary from 5 to 45 dBnHL at the frequency of 500 Hz; from 5 to 40 dBnHL at the frequency of 1000 Hz; from 5 to 45 dBnHL at the frequency of 2000 Hz and from 20 to 50 dBnHL at the frequency of 4000 Hz, with the differences between the frequencies being statistically significant (Fig. 2). To verify the differences between frequency pairs, the Wilcoxon test was performed, which showed a significant difference of the frequency of 500 Hz in relation to the other tested frequencies (*p* = 0.001), and of the frequency of 1000 Hz in relation to the other tested frequencies (*p* = 0.027). No differences were observed between the cortical thresholds at the 2000 and 4000 Hz frequencies. The cortical electrophysiological thresholds by BC were on average: 23.6; 28; 38 and 33.1 dBnHL for the frequencies of 500, 1000, 2000 and 4000 Hz, respectively.

## Discussion

The present study aimed to characterize the cortical auditory evoked potentials by BC so that they can be applied as a clinical diagnostic tool, therefore improving the auditory system assessment tests, especially in children. The auditory evoked potential (AEP) by BC, although recorded and interpreted in the same way as AEP by AC, has some particular advantages. We sought to select the sample of this study according to strict inclusion criteria, with equal distribution in relation to the tested ears, but it was not possible to maintain the distribution according to gender. There was some difficulty finding female subjects for the survey, as only 36% of the recruited subjects attended the test.

Initially, it was necessary to evaluate the question of masking, since there was no agreement between the authors on whether it was necessary or not.^14^, ^15^ However, the results of the present study showed that there was no statistically significant difference in cortical responses in bone conduction with the use of masking, which is justified by the newborn's interaural attenuation, estimated at 25–35 dB. Thus, the collection was completed without the need for contralateral noise use in neonates. Fichino et al.^19^ reported that when performing the BC AABR protocol, they experienced difficulties that they discussed to assist future studies, which were considered in the present study, namely: the bone vibrator emits electromagnetic energy that interferes with the signal detection, and to minimize these artifacts, it is necessary to move the vibrator away from the electrode. The positioning and force of the bone vibrator must be accurate and with a force of 400 ± 25 g, so as not to alter latency time.^20^, ^21^, ^22^ With respect to contralateral masking, they mentioned that interaural attenuation of bone conduction in children younger than 1 year is approximately 35 dB and, therefore, masking is required only at higher intensities. When performing this study, we aimed to ensure a force of ± 400 g, with the bone vibrator attached with a self-adhering elastic band and verified with an Ohaus scale.

Subsequently, the P1 latencies of CAEP AC performed at an intensity of 80 dBnHL were compared with the P1 latencies of the present study, carried out with BC at a maximum intensity of 60 dBnHL, and no statistically significant difference was found. These studies demonstrated that the procedures adopted in the present study allowed a reliable collection. The protocol proposed by Oliveira et al.^6^ was the starting point for the present study, as well as the results reported by Oliveira et al.^6^, who observed that evaluating hearing in neonates through automated capture of CAEP responses by air conduction is effective and feasible, since they found cortical responses in 100% of the tested subjects. In the present study, it was possible to observe cortical responses in 100% of the tested subjects at the intensity of 60 dBnHL, and for the intensity of 35 dBnHL, we found cortical responses in 86% of subjects at frequencies of 500 Hz; 86% at 1000 Hz; 73% at 2000 Hz and 70% at 4000 Hz in the tested subjects. For the intensity of 30 dBnHL, we found cortical responses in 73% of the tested subjects at frequencies of 500 Hz; 66% at 1000 Hz; 53.3% at 2000 Hz and 56.6% 4000 Hz.

We aimed to observe the integrity of the auditory pathway at an intensity of 60 dBnHL, as previous studies have shown that it is possible to find artifacts at 70 dBnHL that hinder the response to the bone vibration transducer.^14^ No significant differences were found in the comparison between the ears. In the analysis of P1 latencies, we found the mean values ​​of: 234 ms for 500 HZ; 241 ms for 1000 Hz; 239 ms for 2000 Hz and 253 ms for 4000 Hz. These findings are similar to those observed by Oliveira et al.,^6^ with latency responses comparable to those found with the air conduction transducer, where P1 is approximately 200–300 ms after acoustic stimulation at 80 dBnHL in normal-hearing newborns.

Regarding the amplitudes of the CAEP P1 component, in this study the values ​​by frequency were 15.6 μV for 500 Hz; 8.4 μV for 1000 Hz; 6.2 μV for 2000 Hz and 6.3 μV for 4000 Hz, with *p* = 0.001. The Wilcoxon test was performed, which showed a significant difference at the frequency of 500 Hz compared to the other tested frequencies (*p* = 0.001). This result agrees with the values ​​found by Small and Stapells,^12^ who compared the results of BC in adults and children in the auditory steady-state response (ASSR) test and the study showed that children are much more sensitive to BC conduction stimuli at low frequencies, and these differences between children and adults persist until at least two years of age. The authors mention several factors that may contribute to this finding, by highlighting the size and structural differences of the skull between the child under 2 years of age and the adult. The child skull is smaller than that of an adult, with smaller dimensions of the mastoid process in width, length and depth. Moreover, there are flexible sutures that connect the temporal bone to the other bones in the child skull, in contrast to the adult skull, which is a rigid structure with fused bones. They also suggested that the flexible sutures of the child skull may result in less energy dissipating to the rest of the skull, causing the temporal bone to oscillate more in isolation, thus resulting in more effective stimulation through the frequencies in children under 1 year of age. They also report that the smaller temporal bone mass in children under 1 year of age results in a stronger signal activating the cochlea.

The thresholds found at specific frequencies by BC in this study were consistent with those found by Oliveira et al.,^6^ who evaluated the CAEP by AC in neonates (Table 4). Both studies used the HEARlab System, with the specific frequency module, the cortical tone evaluation (CTE), to obtain estimates of cortical thresholds at frequencies of 500, 1000, 2000, and 4000 Hz. In another recent study on the same equipment, the authors showed means of electrophysiological responses by AC in adults with normal hearing at 18.23 dBnHL for 500 Hz; 15.9 dBnHL for 1000 Hz; 15.97 dBnHL for 2000 Hz and 17 dBnHL for 4000 Hz, mean values ​​that were lower than those observed in the neonatal studies described in Table 4, probably due to maturational issues.^7^

**Table 4:** Comparison between electrophysiological thresholds and test performance time in relation to Air Conduction (AC) and Bone Conduction (BC) CAEP.

	Média de limiares em dBnNA					
	Type of transducer	500 Hz	1000 Hz	2000 Hz	4000 Hz	Time of examination
Oliveira et al. (2019)^6^	AC	24.87	25	28.72	29.49	1 h 13 m
Present study	BC	23.6	28	31	33.1	1 h 15 m

There is a lack of studies on the use of BC stimulation in AEP. Since no material was found containing information on BC thresholds in CAEP, the use of BC in other types of AEP in the pediatric population was investigated.

Therefore, Table 5 shows the comparison between different estimates of AEP thresholds in normal hearing newborns obtained by BC. The studies by Casey and Small^11^ and Small and Stapell^10^ studies, performed with ASSR, and Elsayed^13^ using AABR with tone burst, were verified. The times presented in the table refer to the evaluation of only one ear.

**Table 5:** Comparison between electrophysiological thresholds obtained by BC and time and examination in different types of AEP in neonates.

Authors	Mean AEP threshold per BC (dBnHL)					
	n	500 Hz	1000 Hz	2000 Hz	4000 Hz	Time of examination
Casey and Small (2014)^11^ ASSR	23	19	20	20	20	2 h
Small and Stapell (2008)^12^ ASSR	35	10	10	40	30	1 h 50 m
Elsayed et al. (2015)^13^ ABR tone burst	145	30	30	25	35	1 h 50 m
Present study	30	20	30	30	30	1 h 15 m

When comparing studies, there is greater agreement with the thresholds obtained in the study performed with AABR.^13^ One can also observe that the duration of the studies by the abovementioned authors is longer than that of the present study. Although studies performed with ASSR^11^, ^12^ obtained lower electrophysiological thresholds than the present one, the time required to perform the test was longer.

The time of evaluation is extremely important for the neonatal population, and the present study demonstrated a similar time to that presented by Oliveira et al.^6^ This study reinforces that automated response capture is a viable and sensitive procedure for the capture of electrophysiological thresholds in the neonatal population, with reduced test performance time.

Another aspect to be emphasized was the higher threshold observed at higher frequencies. The authors Casey and Small^11^ also found higher thresholds at the 2000 and 4000 Hz frequencies. Elsayed et al.^13^ observed higher thresholds at the frequency of 4000 Hz. Small and Stapells^12^ found in their study that children are more sensitive to BC stimuli at lower frequencies, which agrees with the higher amplitudes observed in the present study at 500 Hz.

The sensitivity of cortical response detection in the Hearlab System equipment is high when compared to experienced examiners. Carter et al.^4^ verified the effectiveness of automated response analysis with experienced CAEP examiners and concluded that both the automated analysis equipment and the examiners had high sensitivity in detecting responses.

Several studies addressing the use of different advanced technologies in the auditory system investigation aim to improve the diagnosis in the pediatric population. In normal hearing newborns, failure to obtain otoacoustic emissions is common, due to alterations of the external ear canal and/or middle ear. In these cases, to attain an accurate diagnosis and in addition to behavioral tests, the auditory evoked potentials are used. In these cases, it is extremely important to use the BC in the AEP for the differential diagnosis. Therefore, these new technologies should study the use of tests with sound conduction by BC.

Electrophysiology studies in children with ear malformations^14^, ^23^, ^24^, ^25^ demonstrated higher thresholds through air conduction assessment than bone conduction, which characterizes conductive hearing loss, commonly associated with ear malformations. The electrophysiological thresholds by AC and BC allow the characterization of the audiological profile, which shows a good correlation with the behavioral audiological assessment.^26^ Garcia et al.^25^ warn that it is necessary not to limit the study of children's thresholds to those obtained by air conduction, as it can generate false positives for sensorineural hearing loss. Parry et al.^16^ investigated the CAEP by BC of individuals with unilateral conductive hearing loss and confirmed that it is possible to demonstrate significant alterations in cortical response amplitude in adults with unilateral conductive hearing loss.

The estimate of cortical electrophysiological thresholds by bone conduction were shown to be feasible and could be used to complement the assessment of the auditory system, aiming to contribute to the differential diagnosis in the neonatal population, as well as in individuals who cannot be assessed exclusively through behavioral methods only.

Future studies involving other age groups, as well as individuals with conductive, mixed, sensory, and neural hearing loss, will allow a better understanding of the cortical effects of age and the several types of hearing loss.

## Conclusions

This study allowed the characterization of the CAEP response by BC in normal-hearing neonates using automated response analysis equipment, characterizing P1 component latencies and amplitudes at the intensity of 60 dBnHL and at the cortical threshold.

At the intensity of 60 dBnHL, the P1 component showed the following mean latency values: 234; 241; 239 and 253 ms; and of amplitude: 15.6; 8.4; 6.2; 6.3 μV for the frequencies of 500, 1000, 2000 and 4000 Hz, respectively.

The cortical electrophysiological thresholds by BC were on average: 23.6; 28; 31 and 33.1 dBnHL for the frequencies of 500, 1000, 2000 and 4000 Hz, respectively.

There was no difference in the CAEP response for the variables: gender, ear, and use of masking.

## Conflicts of interest

The authors declare no conflicts of interest.

## References

[1] Melo A., Biaggio E.P.V., Rechia I.C., Sleifer P. (2016). Potenciais evocados auditivos corticais em neonates nascido a termo e pré termo. CoDAS..

[2] Didoné D.D. (2018). Potencial Evocado Auditivo Cortical em nascidos a termo e pré-termo. Universidade Federal do Rio Grande do Sul, Faculdade de Medicina. Programa de Pós-Graduação em Saúde da Criança e do Adolescente..

[3] Van Dun B. HEARLAB technical paper. National Acoustic Laboratories & The HEARing CRC. [online]. July 2017. Avaliable from: http://www.researchgate.net/publication/318663391. Accessed May 15, 2019.

[4] Carter L., Golding M., Dillon H., Seymour J. (2010). The detection of infant cortical auditory evoked potentials (CAEPs) using statistical and visual detection techniques. J Am Acad Audiol..

[5] Didoné D.D., Oliveira L.S., Sleifer P., Riesgo R.S., Garcia M.V., Almeida K. (2018). Effect of the arousal state on automated detection of cortical auditory evoked responses in neonate. Audiol Commun Res..

[6] Oliveira L.S., Didone D.D., Durante A.S. (2019). Automated Cortical Auditory Evoked Potentials threshold estimation in neonates. Braz J Otorhinoloryngol..

[7] Durante A.S., Wieselberg M.B., Roque N., Carvalho S., Pucci B., Gudayol N. (2017). Assessment of hearing threshold in adults with hearing loss using an automated system of cortical auditory evoked potential detection. Braz J Otorhinolaryngol..

[8] Kosaner J., Van Dun B., Yigit O., Gultekin M., Bayguzina S. (2018). Clinically record cortical auditory evoked potentials from paediatric cochlear implant user fitted with electrically elicited stapedius reflex thresholds. Int J Pediatric Otorhinolaryngolory..

[9] Parry L.V., Maslin M.R.D., Schaette R., Moore D.R., Munro K.J. (2019). Increased auditory cortex neural response amplitude in adults with chronic unilateral conductive hearing impairment. Hear Res..

[10] National center for hearing assessment and management – NCHAM. [online] Avaliable from: www.infanthearing.org. Accessed May 15, 2019.

[11] Lewis D.R., Marone S.A.M., Mendes B.C.A., Cruz O.L.M., Nobrega M. (2010). Comitê Multiprofissional em saúde auditiva. COMUSA. Braz J Otorhinolaryngol..

[12] Casey K.A., Small S.A. (2014). Comparisons of auditory steady state response and behavioral air conduction and bone conduction thresholds for infants and adults with normal hearing. Ear Hear..

[13] Small A.S., Stapells D.R. (2008). Multiple auditory steady – state response thresholds to bone- conduction stimuli in young infants with normal hearing. Ear Hear..

[14] Elsayed M.A., Hunter L.L., Keefe D.H., Feeney M.P., Brown D.K., Meinzen-Derr J.K. (2015). Air and Bone conduction click and tone-burst Auditory Brainstem Threshold using Kalman Adaptive Processing in non sedated normal-hearing infants. Ear Hear..

[15] NRPV Curado, Muniz L.F., Silveira A.K., Silva A.R.A., Griz S.M.S. (2015). Bone conducted brainstem auditory evoked response: an integrative review. Rev Cefac..

[16] Lightfoot G. The N1–P2 Cortical Auditory Evoked Potential in threshold estimation. Insights in practice for clinical audiology. [online] February 2010:1-8. Available from: www.audiologyonline.com/articles/article_detail.asp?article_id=2342. Accessed May 15, 2019.

[17] American Academy of Pediatrics, Joint Committee on Infant Hearing (2007). Year 2007 Position Statement: principles and guidelines for early hearing detection and intervention programs. Pediatrics.

[18] Van Dun B., Dillon H., Seeto M. (2015). Estimating hearing threshold in hearing-impaired adults through objective detection of cortical auditory evoked potentials. J Am Acad Audiol..

[19] Fichino S.N., Lewis D.R., Fávero M.L. (2007). Estudo dos limiares eletrofisiológicos das vias aéreas e óssea em crianças com até 2 meses de idade. Rev Bras Otorrinolaringol..

[20] Yang E.Y., Stuard A., Mencher G.T., Mencher L.S., Vincer M.J. (1993). Auditory brainstem responses to air-and –bone- conducted click in the audiological assessment of at-risk infantes. Ear Hear..

[21] Yang E.Y., Stuart A., Stenstrom R., Green W.B. (1993). Test-retest variability of the auditory brainstem response to bone conducted clicks in newborn infants. Audiology..

[22] Stuart A., Yang E.Y., Stenstrom R., Reindorp A.G. (1993). Auditory Brainstem response thresholds to air and bone conducted clicks in neonates and adults. Am J Otol..

[23] Chen W.X., Wang Y., Lu P., Huang Y., Xu Z.M. (2015). Air and bone conduction auditory brainstem response in children with congenital external auditory canal atresia. Arch Otolarinyngo Rhinol..

[24] Sleifer P., Didone D.D., Keppeler I.B., Bueno C.D., Riesgo R.D.S. (2017). Air and bone conduction frequency-specific auditory brainstem response in children with agenesis of the external auditory canal. Int Arch Otorhinolaryngol..

[25] Garcia M.V., Azevedo M.F., Biaggio E.P.V., Didoné D.D., Testa J.R.G. (2014). Potencial evocato auditivo de Estado Estável por via aérea e via óssea em crianças de zero a seis meses sem e com comprometimento condutivo. Rev CEFAC..

[26] BC Early Hearing Program (2012).

